# Supplementary material for: Spatial distribution pattern and driving mechanism of tourist attractions in Gansu Province based on POI data
Source: PLoS One. 2023 Oct 5;18(10):e0292165. doi: 10.1371/journal.pone.0292165 (PMC10553315; doi:10.1371/journal.pone.0292165)
Supplement: Attachment — Submitted filename: Reviewer comments23.5.9.docx [file pone.0292165.s001.docx]

**Spatial distribution pattern and driving mechanism of tourist attractions in Gansu Province based on POI data**

**Comments**

Based on the POI data, this paper analyzes the spatial distribution pattern of various types of tourist attractions with the spatial autocorrelation analysis and kernel density analysis methods in Gansu province, and probes into the driving mechanism. This study provides a scientific basis for the future development and layout of tourist developments and helps the tourism industry to develop with high quality in Gansu province. However, there are a number of major issues that the authors have to carefully consider in this manuscript:

1. The research significance or academic contribution of this article in the Introduction is insufficient. The authors only introduces the role and value of this study for Gansu province.
2. In the Part 3.1, the analysis focuses on the results of different methods rather than the spatial distribution of various types of tourist attractions. Moreover, this part only classifies the distribution of tourist attractions, without specifying the size and grade of the attractions, or explaining the causes of spatial distribution pattern.
3. In the Part 3.2.1, the analysis of these driving factors is only objective and general facts, which are weakly related to the spatial distribution of tourist attractions in Gansu, especially the natural environment level and level of transportation facilities. Some analyses and explanations are irrelevant or even wrong. For example, Gannan Prefecure and Jinchang City and other areas have not formed adequate road transportation system, and the tourist attractions are scattered. Does it imply that the scattered tourist attractions are due to poor road transportation system? In addition, it does not make sense that tourism service facilities and service personnel affect the formation of spatial distribution of tourist attractions.
4. In the Part 3.2.2, it is not logical to analyze the driving factors of the spatial distribution pattern of tourist attractions from the perspectives of social economy, transportation facilities and tourism services. The spatial distribution of tourist attractions, especially natural attractions, is the result of natural and geological action, and will not be more or less distributed due to social economy and tourism services. The star hotels, the number of tourists, and the number of accommodation and catering workers are not the driving factors of spatial distribution pattern of tourist attractions.
5. In the Part 3.2.3, GDP per capita, resident population, total road mileage, private car ownership and bus ownership are not the driving force of spatial distribution of tourist attractions.

In short, there are some major problems in the current paper, such as some deviation in its intention, insufficient logical argument, analysis of influencing factors instead of driving mechanism, etc., and the results and conclusions are not credible enough.
