# Supplementary material for: Spatial distribution pattern and driving mechanism of tourist attractions in Gansu Province based on POI data
Source: PLoS One. 2023 Oct 5;18(10):e0292165. doi: 10.1371/journal.pone.0292165 (PMC10553315; doi:10.1371/journal.pone.0292165)
Supplement: Attachment — Submitted filename: Response to Reviewers.docx [file pone.0292165.s002.docx]

**Dear Editor,**

**We sincerely thank the editor and all reviewers for their valuable feedback that we have used to improve the quality of our manuscript. There reviewer comments are laid out below in italicized font and specific concerns have been numbered. Our response is given in normal font.**

**Sincerely,**

**Wanqianrong Gao, Master's degree**

**Tourism College of Northwest Normal University**

**730070, 13993813451,** [gaowanqianrong@163.com](mailto:gaowanqianrong@163.com)

***Editor comments:***

1. *The research question of your study is unclear. I suggest that you clarify these aspects of your research to improve the manuscript's overall quality.*

We sincerely thank the reviewer for careful reading. As suggested by the reviewer. We have sorted out the research questions and defined them more clearly. This is reflected in the blue font of the abstract and introduction.

2. *Furthermore, several key pieces of literature on spatial autocorrelation analysis are missing. It is essential to include relevant and influential literature to establish the context of your research and demonstrate a comprehensive understanding of the subject matter. Please ensure that you include the key literature, such as Anselin (1988) on Spatial Econometrics and Anselin (1995) on Local Indicators of Spatial Association (LISA).*

We sincerely appreciate the valuable comments. We have checked the literature carefully and added key literature on spatial econometrics and on local indicators of spatial associations to the methods section of the manuscript. (Reference 29, 30, 31, 32, 33, 34, 35)

3. *Finally, I would like to mention that the language used in the paper is challenging to understand. There are several unnecessarily lengthy sentences that could be simplified for clarity.*

Thanks for your suggestion. We have tried our best to polish the lan guage in the revised manuscript. Long sentences in the introduction and conclusion have been simplified.

4. *During our internal evaluation of the manuscript, we found significant text overlap between your submission and previous work in the [introduction, conclusion, etc.].*

We sincerely thank the reviewer for careful reading. We have revised the introduction and conclusion sections of the manuscript and introduced sources.

5. *We note that Figure 1, 2, 4, 5 in your submission contain [map/satellite] images which may be copyrighted.*

The data sources in this paper are all openly available, and in addition, we have made changes to the data sources section of the revised version to explain the changes based on the revisions. And the naming of the images is explained in detail.

Image captions updated:

Fig 1. Geographical location of Gansu Province and distribution map of various cities and cities. Contains data from Contains information from National Geomatics Center of China (https://www.ngcc.cn/ngcc/html/1/391/392/16114.html) and Resource and Environmental Science Data Center of Chinese (https://www.resdc.cn/data.aspx?DATAID=123), they are freely available.

Fig 2. Distribution map of tourist attractions in Gansu Province. Contains data from Contains information from National Geomatics Center of China (https://www.ngcc.cn/ngcc/html/1/391/392/16114.html), they are freely available.

Fig 4. LISA agglomeration figure of various types of tourist attractions in Gansu Province. Contains data from Contains information from National Geomatics Center of China (https://www.ngcc.cn/ngcc/html/1/391/392/16114.html), they are freely available.

Fig 5. Kernel density of various tourist attractions in Gansu Province. Contains data from Contains information from National Geomatics Center of China (https://www.ngcc.cn/ngcc/html/1/391/392/16114.html), they are freely available.

Fig 7. Distribution of Tourist Attractions in Gansu Province Coupled with Topography and River Buffers. Contains data from Contains information from National Geomatics Center of China (https://www.ngcc.cn/ngcc/html/1/391/392/16114.html) and Resource and Environmental Science Data Center of Chinese (https://www.resdc.cn/data.aspx?DATAID=123), they are freely available.

Fig 8. Distribution of tourist attractions in Gansu Province traffic and urban buffer zone. Contains data from Contains information from National Geomatics Center of China (https://www.ngcc.cn/ngcc/html/1/391/392/16114.html) and Resource and Environmental Science Data Center of Chinese (https://www.resdc.cn/data.aspx?DATAID=123), they are freely available.

***Review 1 Comments:***

1. *It is recommended to use Chinese standard maps with review numbers for maps involving national boundaries.*

Thanks for your suggestion. We have carefully read the journal's graphic requirements and have completed the changes as required.

2. *Try to use data from 2022 as much as possible.*

Thanks for your suggestion. Our data was collected at the end of December 2021, and the paper was completed in 2022. As the data is the basis of the article, it is too much work to change the data, so we ask the reviewers to understand and sympathize.

3. *For literature citations, please use English citation standards such as APA format according to the journal's requirements.*

We were really sorry for our careless mistakes. Thank you for your reminder. We have changed the citation to the English literature citation standard.

***Review 2 Comments:***

1. *The research significance or academic contribution of this article in the Introduction is insufficient. The authors only introduces the role and value of this study for Gansu province.*

We sincerely thank the reviewer for careful reading. We have deepened the relevant elements, as reflected in the concluding part of the introduction.

2. *In the Part 3.1, the analysis focuses on the results of different methods rather than the spatial distribution of various types of tourist attractions. Moreover, this part only classifies the distribution of tourist attractions, without specifying the size and grade of the attractions, or explaining the causes of spatial distribution pattern.*

Thanks for your suggestion. We have revised the section to sort out the tourist attraction classes and explain the reasons for the spatial distribution pattern.

3. *In the Part 3.2.1, the analysis of these driving factors is only objective and general facts, which are weakly related to the spatial distribution of tourist attractions in Gansu, especially the natural environment level and level of transportation facilities. Some analyses and explanations are irrelevant or even wrong. For example, Gannan Prefecure and Jinchang City and other areas have not formed adequate road transportation system, and the tourist attractions are scattered. Does it imply that the scattered tourist attractions are due to poor road transportation system? In addition, it does not make sense that tourism service facilities and service personnel affect the formation of spatial distribution of tourist attractions.*

We sincerely appreciate the valuable comments. We have revised the section and re-selected the drivers that shape the spatial pattern of tourist attractions, which are now analyzed in terms of the natural environment, location and transportation, and socio-economics.

4. *In the Part 3.2.2, it is not logical to analyze the driving factors of the spatial distribution pattern of tourist attractions from the perspectives of social economy, transportation facilities and tourism services. The spatial distribution of tourist attractions, especially natural attractions, is the result of natural and geological action, and will not be more or less distributed due to social economy and tourism services. The star hotels, the number of tourists, and the number of accommodation and catering workers are not the driving factors of spatial distribution pattern of tourist attractions.*

We sincerely appreciate the valuable comments. We have made changes to the selection of drivers.

5. *In the Part 3.2.3, GDP per capita, resident population, total road mileage, private car ownership and bus ownership are not the driving force of spatial distribution of tourist attractions.*

We sincerely appreciate the valuable comments. We have removed the drivers you mentioned above from the manuscript.

***Review 3 Comments:***

*In this study, the analysis investigating the spatial influences applied inappropriate methods. Thus, the main findings of this study are overstated. This issue is critically considered as the serious flaw of this paper. It is noted that conventionally, the spatial analysis employs spatial regression techniques (e.g., spatial lag model, spatial error model, spatial Durbin model) for quantifying the spatial spillovers. By integrating the GIS framework and the statistical inference, the spatial regression can systematically identify the relationship among variables on geographical space.*

We sincerely appreciate the valuable comments. We changed the geodetector approach to quantitative analysis of drivers to OLS modeling in buffer analysis and spatial autoregressive analysis.

We tried our best to improve the manuscript and made some changes marked in blue in revised paper which will not influence the content and framework of the paper. We appreciate for Editors and Reviewers’warm work earnestly, and hope the correction will meetwith approval.Once again, thank you very much for your comments and suggestions.
